# Effective Endotoxin Removal from Chitosan That Preserves Chemical Structure and Improves Compatibility with Immune Cells

**DOI:** 10.3390/polym15071592

**Published:** 2023-03-23

**Authors:** Sophie L. Reay, Emma L. Jackson, Daniel Salthouse, Ana Marina Ferreira, Catharien M. U. Hilkens, Katarina Novakovic

**Affiliations:** 1 School of Engineering, Newcastle University, Newcastle upon Tyne NE1 7RU, UK; 2 Translational & Clinical Research Institute, Newcastle University, Newcastle upon Tyne NE2 4HH, UK

**Keywords:** chitosan, endotoxin removal, alkali treatment, heat treatment, Maillard reaction, pro-inflammatory response, monocyte-derived dendritic cells, lysozyme degradation

## Abstract

Chitosan is one of the most researched biopolymers for healthcare applications, however, being a naturally derived polymer, it is susceptible to endotoxin contamination, which elicits pro-inflammatory responses, skewing chitosan’s performance and leading to inaccurate conclusions. It is therefore critical that endotoxins are quantified and removed for in vivo use. Here, heat and mild NaOH treatment are investigated as facile endotoxin removal methods from chitosan. Both treatments effectively removed endotoxin to below the FDA limit for medical devices (<0.5 EU/mL). However, in co-culture with peripheral blood mononuclear cells (PBMCs), only NaOH-treated chitosan prevented TNF-α production. While endotoxin removal is the principal task, the preservation of chitosan’s structure is vital for the synthesis and lysozyme degradation of chitosan-based hydrogels. The chemical properties of NaOH-treated chitosan (by FTIR-ATR) were significantly similar to its native composition, whereas the heat-treated chitosan evidenced macroscopic chemical and physical changes associated with the Maillard reaction, deeming this treatment unsuitable for further applications. Degradation studies conducted with lysozyme demonstrated that the degradation rates of native and NaOH-treated chitosan-genipin hydrogels were similar. In vitro co-culture studies showed that NaOH hydrogels did not negatively affect the cell viability of monocyte-derived dendritic cells (moDCs), nor induce phenotypical maturation or pro-inflammatory cytokine release.

## 1. Introduction

**Figure 1:**
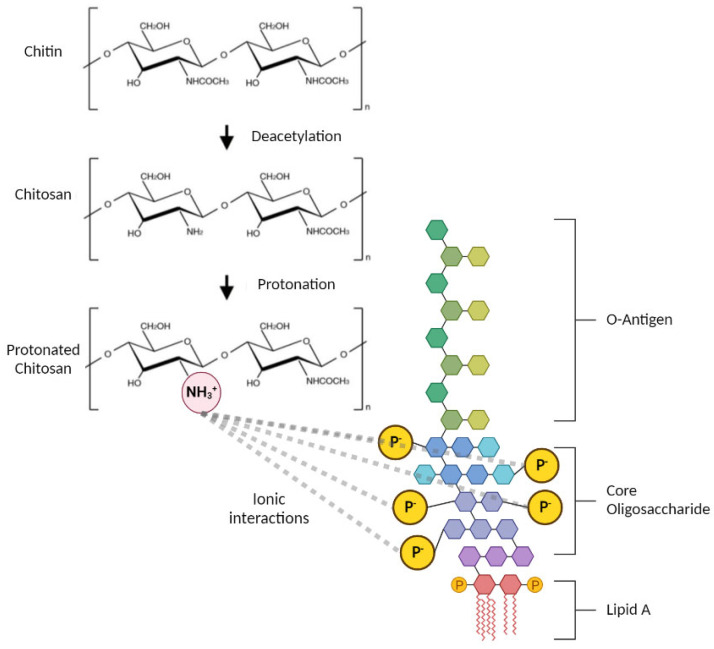
Structure of chitosan and LPS and their ionic interaction (created with BioRender, adapted from [24]).

Chitosan is a linear, cationic copolymer comprised of randomly distributed β-(1,4)-linked N-acetyl-D-glucosamine and D-glucosamine units. It is produced from the deacetylation of chitin (Figure 1) which is in fungal cell walls and crustaceans, with the latter being a major waste product of the fishing industry [1,2,3]. In the human body, chitosan is predominantly degraded by lysozyme, which only interacts with the acetylated units of chitosan [4,5,6]. Given that chitosan is abundant, renewable, biocompatible, biodegradable, non-toxic, and anti-microbial [7,8,9], it is clear why chitosan is of utmost interest in high-value applications. Chitosan has been designated ‘Generally Recognized As Safe (GRAS)’ by the FDA and is approved for use in dietary supplements [10] as well as in biomedical applications, such as wound dressings and gels [11,12]. Importantly, chitosan is one of the leading materials studied for central nervous system applications because of its ability to cross the blood brain barrier [13]. Furthermore, the expanding interest in this material is evidenced by the growing worldwide chitosan market, which is expected to grow to $12.14 billion in 2026 at a compound annual growth rate of 19.2% [14].

A major issue that needs to be considered for any biomedical in vivo application of chitosan is the contamination with bacterial lipopolysaccharides (LPS), also known as endotoxins. These are heat-stable molecules (<180 °C) found in the outer membrane of Gram-negative bacteria [15,16,17], and are composed of three parts: lipid A, a core oligosaccharide, and an O-antigen (Figure 1). The inner core is highly phosphorylated, causing LPS to be anionic [18,19]. Innate immune cells recognize the lipid A portion of endotoxin through Toll-like receptor-4 (TLR4), which initiates downstream signalling leading to proinflammatory cytokine release, resulting in activation of potent immune responses [20]. The host immune system can detect and elicit potent proinflammatory responses in response to extremely small concentrations of endotoxin [21]; for example, monocytes and dendritic cells are shown to be activated by endotoxin concentrations as low as 0.01 ng/mL (0.05 EU/mL) [15] and 0.02 ng/mL (0.1 EU/mL) [22], respectively. The FDA have therefore enforced strict regulations on endotoxin levels in medical devices (limit of 0.5 EU/mL [23]), with devices continually being recalled due to their endotoxin levels exceeding the limit [17].

Many studies employ natural sources of biomaterials, which are susceptible to endotoxin contamination [25,26]. Furthermore, synthetic biomaterials can also be contaminated with endotoxin due to contaminated laboratory equipment or reagents [15,17]. Endotoxin contamination is a particular concern for chitosan as the cationic nature of the polymer predisposes its interaction with the negatively charged phosphate groups in LPS (Figure 1) [27]. Despite the continuous increase in the number of scientific publications related to chitosan [28], the purity levels, and in particular endotoxin contamination, are seldom acknowledged [21,29]. Chitosan is renowned for its potent immunostimulatory ability [30,31,32], and has been reported to elicit both pro- and anti-inflammatory responses [33,34,35,36]. Furthermore, there are contrasting findings concerning the cytotoxicity of chitosan [37,38,39]. As endotoxins have inflammatory and cytotoxic effects [40], variable endotoxin contamination could be the reason behind such divergent reports in the literature [41]. Unless endotoxin is quantified and removed, bioassay studies of chitosan (or any other biomaterial) are incomplete and prone to misleading conclusions, limiting their validity, and obstructing intended translation into clinical practice.

Several methods have been developed to remove endotoxin from biomaterials. For example, ultrafiltration and size-exclusion chromatography remove endotoxin based on size. Although endotoxins are approximately 10 kDa, they readily form large aggregates up to 1000 kDa [42,43], making these methods difficult. Moreover, most commercially available endotoxin removal resins combine porous cellulose beads for the matrix and cationic poly(ε-lysine), as the affinity ligand. Examples include the Pierce™ High-Capacity Endotoxin Removal Resin and Cellufine™ ETclean. However, endotoxin removal efficiency is dramatically reduced in viscous and positively charged samples [44], namely chitosan. Ultrasonication and two-phase extraction using detergents are also potential endotoxin removal methods; however, these methods can damage biomaterials, negating their performance [17]. Less complex methods to remove endotoxins from chitosan involve the use of heat and alkali treatments. Previous studies investigating heat treatment found that temperatures of 180 °C or above can destroy endotoxin [15,16]. Heat treatment is often disregarded as an endotoxin removal method, due to the thermal stability of endotoxins [15,16,17]; however, chitosan degradation temperature is reported to be approximately 300 °C [45,46,47,48], therefore chitosan should theoretically withstand these temperatures without compromising its structure. Similarly, the use of alkali treatments such as NaOH has shown promising results in inactivating endotoxins in chitosan due to the hydrolysis of ester and amide linkages found in the lipid A portion [49,50]. Starting from highly deacetylated (95%) and extensively purified ChitoClear^®^ chitosan, Lebre and colleagues used acid-alkaline treatment to remove endotoxin [41]. It is important to note that the presence of acetyl groups is vital for in vivo lysozyme degradation of chitosan-based hydrogels [4,5,6]. If endotoxin removal methods lead to a significant increase in deacetylation degree (DD), they are not suitable for the purification of chitosan intended to degrade in vivo.

This study investigates the suitability of heat and NaOH treatment for the removal of endotoxin from commonly used Sigma-Aldrich medium molecular weight chitosan. For this, chitosan endotoxin levels are quantified with Pierce™ Chromogenic Limulus Amebocyte Lysate (LAL) assay, while the effectiveness of endotoxin removal is also investigated via an In vitro TNF-α bioassay. Fourier-transform infrared (FTIR) spectroscopy was employed to determine the effect of endotoxin removal methods on chitosan structure and change in DD. The ability of treated materials to form chitosan-genipin hydrogels for further biomedical applications is validated, and subsequently, lysozyme degradation of resultant hydrogels was monitored. Chitosan-genipin hydrogels were finally cultured with monocyte-derived dendritic cells (moDCs) to assess cytocompatibility.

## 2. Materials and Methods

### 2.1. Materials

Chitosan (medium molecular weight 190,000–300,000 gmol^−1^, 75–85% deacetylation, product code 448877, lot number STBG5137V), glacial acetic acid (product code 101884980, lot number STBH0491), genipin (≥98%, product code G4796, lot number 0000111438), 1.0 M NaOH solution (product code S2770-100ML, lot number RNBK6726), Roswell Park Memorial Institute (RPMI) 1649 media, L-glutamine, penicillin/streptomycin, polymyxin B and O-Phenylenediamine dihydrochloride were supplied from Sigma-Aldrich (Irvine, UK). Foetal calf serum was supplied by Gibco. IL-4 was supplied by Miltenyi Biotec (Bergisch Gladbach, Germany) and GM-CSF was supplied by Genzyme (Cambridge, MA, USA). Endotoxin-free water was purchased from EMD Milipore (Darmstadt, Germany). Human TNF-alpha DuoSet ELISA kit was purchased from R&D Systems (Minneapolis, MN, USA). Lymphoprep was purchased from StemCell (Bernburg, Germany).

### 2.2. Heat Treatment for Endotoxin Removal

Chitosan powder (2 g) contained in a crucible was heated in a pre-heated Clifton drying oven at temperatures of 120, 140, 160 and 180 °C for 0.75, 1.5 and 3 h. The conditions of 180 °C for 3 h were selected as they have been reported to destroy endotoxin [15,16], while lower temperatures and durations were chosen to evaluate the feasibility of endotoxin removal with milder conditions.

### 2.3. NaOH Treatment for Endotoxin Removal

Chitosan powder (1 g) was mixed with 20 mL of 1.0 M NaOH solution using a magnetic stirrer. Conditions were based on reports that NaOH hydrolyses the ester and amide linkages of the lipid A portion in endotoxin [49,50]. The mixture was stirred at room temperature for 2 h, 24 h, 2 h with the addition of polymyxin B (final concentration of 100 μg/mL) as well as 50 °C for 2 h. Polymyxin B was added as a high-affinity LPS ligand [51,52], which was postulated to improve endotoxin removal. Each mixture was filtered with a 70 μm cell strainer to remove the solution. The resultant wet chitosan powder was washed with endotoxin-free water until the pH of the filtrate was 7, and subsequently transferred to an ethanol-sterilised open container and dried in an ethanol-sterilised Clifton drying oven at 37 °C for 24 h.

### 2.4. Preparing Chitosan Solutions

Chitosan solutions (1.5% *w*/*v*) were prepared by dissolving native, heat-treated (180 °C for 1.5 h) and NaOH-treated (1.0 M NaOH for 2 h) chitosan powders in acetic acid solution (1% *v*/*v*). The solutions were stirred with a magnetic stirrer in sealed vessels for 24 h to obtain pale yellow, viscous solutions.

### 2.5. PBMC Isolation and Culture

Leukocyte Reduction System cones were obtained from healthy donors with informed consent and ethical approval from the Faculty of Medical Sciences Ethics Committee, Newcastle University (1659/10369/2019), Newcastle upon Tyne, UK. Whole blood was obtained from healthy donors with informed consent and ethical approval from the Animal Welfare and Ethical Review Body, Newcastle University (AWERB Project ID No: ID 633). Peripheral blood mononuclear cells (PBMCs) were isolated by density centrifugation on Lymphoprep. Chitosan solution was prepared following the method outlined in Section 2.4. The wells of a 24-well plate (Costar, Deeside, UK) were coated with 200 µL chitosan solution. Plates were dried for 24 h in a TriMat 2 microbiological safety cabinet to produce chitosan films. As chitosan solution is slightly acidic, dried films were neutralised with 0.5 mL 0.1 M NaOH solution and subsequently washed five times with 1 mL Hanks Balanced Salt Solution (HBSS) containing phenol red indicator until the colour of the solution changed to red, suggesting a neutral pH and complete removal of excess NaOH.

#### 2.5.1. CLI-095 Culture

PBMCs (1 × 10^6^/mL) were suspended in RF10 media (RPMI 1640 substituted with 1% penicillin-streptomycin, 2% glutamine and 10% fetal calf serum) +/− the TLR4 inhibitor CLI-095 (5 µg/mL) in 15 mL falcon tubes and incubated at 37 °C with 5% CO_2_ for 6 h on a MACSmix™ Tube Rotator (Miltenyi Biotec, Bergisch Gladbach, Germany). PBMCs were then added to an uncoated or chitosan-coated 24-well tissue culture plate (1 × 10^6^ PBMCs per well) and further incubated for 18 h. Supernatant (200 µL) was removed and frozen at −80 °C for determination of cytokine secretion by TNF-α ELISA (Section 2.6).

#### 2.5.2. TNF-α Bioassay

Native, heat-treated and NaOH-treated chitosan films were prepared. PBMCs (1 × 10^6^/mL) were suspended in RF10 media and added to the prepared 24-well plate with or without 100 ng/mL LPS and incubated at 37 °C with 5% CO_2_ for 24 h. Two hundred (200) μL of supernatant was removed and frozen at −80 °C for determination of cytokine secretion by TNF-α ELISA (Section 2.6).

### 2.6. ELISA

Supernatants from the cellular experiments were analysed by TNF-α sandwich enzyme-linked immunosorbent assay (ELISA). Briefly, a 96-well plate was prepared with 4 µg/mL capture antibody overnight. The plate was washed with PBS + 0.1% Tween 20 and supernatant was diluted in PBS + 1% BSA and added to the plate for 2 h. Following washing, 50 ng/mL detection antibody was added for 2 h. The washing step was repeated and Streptavidin-HRP was added to the plate for 30 min before the plate was washed again. OPD was dissolved in citrate phosphate buffer (26.5 mM citric acid, 51.6 mM Na_2_HPO_4_, 51.5 mM Na_2_HPO_4_·2H_2_O) and H_2_O_2_, which was added for 15–30 min until the colour developed. Next, 3 M H_2_SO_4_ was added as a stop solution. The optical density was measured immediately using a Tecan Sunrise™ Absorbance Microplate Reader at 490 nm. The concentration of cytokine in the cell culture supernatants was determined by interpolation of the endotoxin standard curve, using Microsoft Excel software.

### 2.7. Endotoxin Quantification

The endotoxin content of the native and treated chitosan was determined using the Pierce™ Chromogenic Endotoxin Quantification Kit (Limulus Amebocyte Lysate [LAL] assay), following the manufacturer’s protocol. First, chitosan powders were suspended in endotoxin-free water overnight to mimic making a 1.5% *w*/*v* mixture. Chitosan powder suspended in endotoxin-free water was used rather than chitosan solution (typically prepared with the aid of acetic acid [53,54,55]), as chitosan solution is viscous and prevents homogeneous mixing of LAL reagents. Furthermore, chitosan solution is typically yellow, and the LAL assay measures yellow products photometrically at 405 nm, which may affect readings. Supernatants were then pipetted into a 24-well plate that was pre-heated and maintained at 37 °C using a Mixer HC (Starlab, Milton Keynes, UK). Reconstituted Amebocyte Lysate Reagent was added, followed by reconstituted chromogenic substrate solution and 25% acetic acid stop solution at specified time points. The optical density at 405 nm was immediately measured after assay completion using the Tecan Sunrise™ Absorbance Microplate Reader. The developed colour intensity is proportional to the amount of endotoxin present in the sample and was calculated using a standard curve.

### 2.8. Synthesis of Chitosan-Genipin Hydrogel Films

Chitosan solutions were prepared following the method in Section 2.4. Genipin solution (1% *w*/*v*) was prepared by dissolving genipin powder in endotoxin-free water. Chitosan solution (2 mL of 1.5% *w*/*v*) was mixed for 3 min with genipin solution (0.4 mL of 1% *w*/*v*) using a magnetic stirrer. Chitosan-genipin mixture (500 μL) was then transferred to Vision Plate™ 24 microplate, sealed with a plastic cover and hydrogel films were formed in a Clifton drying oven at 37 °C for 24 h.

### 2.9. Synthesis of Chitosan-Genipin Hydrogel Disks

Chitosan and genipin solutions were prepared following the methods outlined in Section 2.4 and Section 2.8. Chitosan solution (1 mL, 1.5% *w*/*v*) was mixed with genipin solution (0.2 mL, 1% *w*/*v*) in a sealed polyethylene vial (15 mm diameter) and placed in a Clifton drying oven at 37 °C for 24 h to form chitosan-genipin hydrogel disks.

### 2.10. FTIR

FTIR spectroscopy was conducted to determine if heat or NaOH treatment induces chemical changes to native chitosan powder. An Agilent Technologies Cary 630 FTIR spectrometer (Agilent, Santa Clara, CA, USA) in attenuated total reflection (ATR), equipped with diamond crystal, was used to obtain FTIR spectra for samples between 4000 and 650 cm^−1^ in transmittance mode. Thirty-two (32) background scans were taken before 64 sample scans. DD of native and NaOH-treated chitosan was calculated from the spectra using Equation (1) [56]. In the equation, A1320 and A1420 represent the transmission at 1320 cm^−1^ and 1420 cm^−1^, respectively.

(1) $$Deacetylation\;degree\;\left(\%\right)=100-\left(\frac{A1320}{A1420}-0.3822\right)\times 1/0.03133\;$$

### 2.11. Lysozyme Degradation

The lysozyme degradation of chitosan-genipin hydrogel disks (prepared following the method outlined in Section 2.9) was investigated gravimetrically in lysozyme/PBS solution containing 2 mg/mL lysozyme. The concentration, higher than physiological, was selected to enhance degradation and reduce experimental time [6]. An empty 70 µm cell strainer was first weighed. Formed hydrogels were then removed from vials, transferred to a cell strainer, and weighed. Hydrogels were then immersed into 30 mL of PBS/lysozyme solution and their weight was recorded at regular time points, after draining solution from the strainer and removing excess solution from the hydrogel surfaces using filter paper. The experiment was performed in triplicate. The degradation rate was calculated using Equation (2), where *W*0 is the initial weight of the sample and *Wd* is the weight of the sample following immersion in PBS/lysozyme solution.

(2) $$Degradation\;rate\;\left(\%\right)=\left[\frac{W0-Wd}{W0}\right]\times 100$$

### 2.12. moDC Isolation and Co-Culture Experiments

Monocytes were isolated from PBMCs using CD14+ microbeads with a MACS separation column (Miltenyi Biotech, Woking, UK). CD14+ monocytes were cultured at 0.5 × 10^6^ cells/mL in a 24-well plate in RF10 media with GM-CSF and IL-4 (both at 50 ng/mL) for 6 days to generate immature moDCs. Media was refreshed on day 3 with GM-CSF and IL-4. On day 6 of moDC generation protocol, ~0.24 g chitosan-genipin hydrogels were added to the cell culture alongside 100 µL of RF10 media with or without LPS to achieve a final concentration of 100 ng/mL. The negative control was supplemented with RF10 only and the positive control with RF10 with LPS. Cells were incubated at 37 °C with 5% CO_2_ for 24 h, after which 200 µL of supernatant was removed and frozen at −80 °C for determination of cytokine secretion by ELISA, and cells were harvested and assessed for expression of cell surface markers by flow cytometry.

### 2.13. Flow Cytometry

Harvested moDCs were suspended in FACS buffer (PBS + 0.5% BSA + 1 mM EDTA + 0.01% sodium azide). The moDCs were incubated with the viability dye, zombie aqua. moDCs were then incubated with fluorescently labelled monoclonal antibodies to cell surface markers of interest (mAbs) (Supplementary Table S1) and human IgG (200 µg/mL) to prevent Fc receptor binding of the mAbs. The cells were then suspended in binding buffer (0.01 M Hepes (pH 7.4), 0.14 M NaCl and 2.5 mM CaCl_2_) and Annexin V was added to detect apoptotic cells. Cells were fixed in 1% formaldehyde and acquired on the Fortessa X20; data were analysed using FCS Express (version 7.16.0047, DeNovo software, Los Angeles, CA, USA). The flow cytometry gating strategy can be found in Supplementary Figure S1. BD Biosciences Anti-Mouse Ig, k/Negative Control Compensation Particles were used for fluorescence compensation settings.

### 2.14. Statistical Analyses

Statistical analyses for most experiments were performed using GraphPad Prism software, version 9.5.0 (730), GraphPad Software Inc., La Jolla, CA, USA. The data are presented as means ± standard deviation and were compared using either the Student’s t-test or ANOVA and Tukey post hoc tests at 95% confidence level. A one-tailed binomial test was used to test for statistical significance in TNF-α production from PBMCs cultured on chitosan films +/− CLI-095 and moDCs cultured with or without NaOH chitosan-genipin hydrogels. PBMC TNF-α bioassay values were analysed using a general linear model separating batch, donor and treatment effects using JMP software. There were significant differences between treatment groups and so multiple comparisons were performed to compare groups using Tukey’s correction for multiple pairwise tests. *p* values less than 0.05 were considered significant.

## 3. Results and Discussion

### 3.1. Endotoxin Contamination of Chitosan

**Figure 2:**
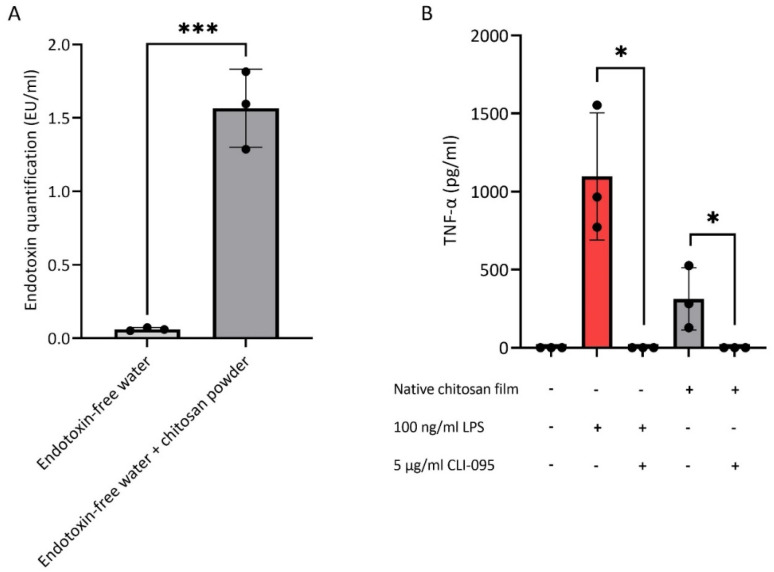
(**A**) Endotoxin quantification of native chitosan powder suspended in endotoxin free water determined by LAL assay. N = 3. (**B**) TNF-α production of PBMCs cultured on native chitosan films or with 100 ng/mL LPS, with or without the TLR4 small molecule inhibitor, CLI-095, for 18 h. PBMCs cultured on untreated plate served as negative control. (+) indicates included and (-) not included compounds. N = 3 (black circles indicate repeats); * *p* < 0.05; *** *p* < 0.001 (black comparator lines).

The LAL assay was first conducted to measure the levels of endotoxin in native chitosan samples incubated in endotoxin-free water. As shown in Figure 2A, endotoxin levels exceeded the 0.5 EU/mL FDA limit for medical devices [23]. Furthermore, to confirm In vitro the presence of endotoxin in chitosan, PBMCs were cultured on chitosan films and the production of the pro-inflammatory cytokine, TNF-α, was measured (Figure 2B). Endotoxin is a known pathogen-associated molecular pattern that signals through Toll-like receptor (TLR)4 expressed by immune cells [57]. The specific small molecule inhibitor of TLR4 signalling, CLI-095, was used to assess whether TNF-α induction by chitosan was mediated through this receptor. Indeed, blocking of TLR4 signalling completely abrogated the secretion of TNF-α (Figure 2B). Together, these data indicate that native chitosan is contaminated with sufficient endotoxin levels to induce pro-inflammatory cytokine production by immune cells in a TLR4-dependent manner.

### 3.2. Endotoxin Removal from Chitosan

**Figure 3:**
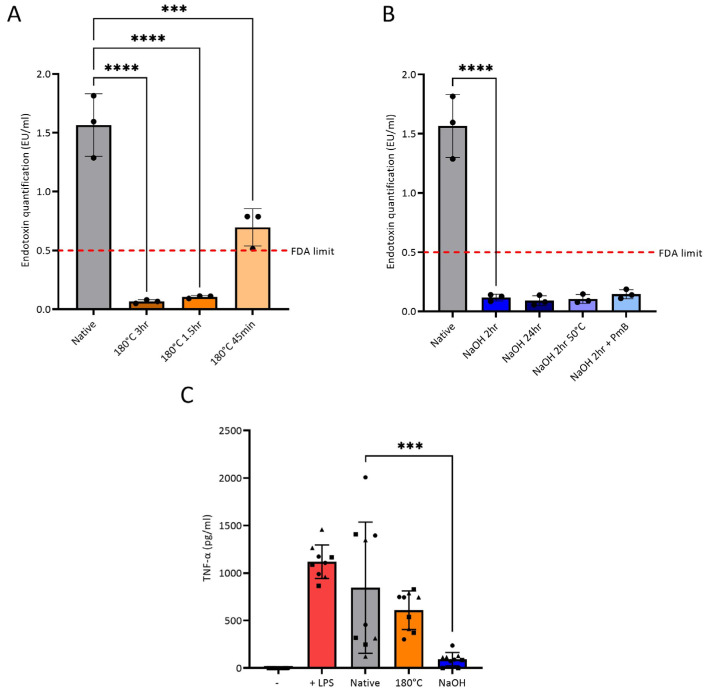
Endotoxin quantification of chitosan samples treated at 180 °C (**A**) or with NaOH (**B**) determined by the LAL assay. N = 3 (black circles indicate repeats). (**C**) TNF-α production by PBMCs from three donors cultured on films synthesized from three batches of each optimal treatment type (180 °C for 1.5 h or NaOH for 2 h) versus native chitosan for 24 h. PBMCs were cultured alone with or without 100 ng/mL LPS as a positive or negative control, respectively. TNF-α values were analysed for statistical significance using a general linear model separating batch, donor and treatment effects. There were significant differences between treatment groups and multiple comparisons were performed to compare groups using Tukey’s correction for multiple pairwise tests. N = 9 (each symbol represents a different batch of chitosan solution used to make films). *** *p* < 0.001; **** *p* < 0.0001.

Two methods of endotoxin removal from chitosan were explored: heat treatment and NaOH treatment. Native chitosan powder was exposed to 120, 140, 160 and 180 °C for 0.75, 1.5 and 3 h. Temperature of 180 °C and durations were based on reports of this temperature being sufficient to destroy endotoxin [15,16]. Differential scanning calorimetry (DSC) was first conducted to determine if heat treatment is a suitable endotoxin removal method for chitosan (Supplementary Method S1.1). The DSC thermographs of chitosan powder showed that it has a degradation temperature of approximately 303 °C (Supplementary Figure S2), which is in agreement with the literature [45,46,47,48], suggesting that the polymer is stable at 180 °C. While temperatures below 180 °C did not suffice in bringing endotoxin level below FDA-approved limit of 0.5 EU/mL [23] (results not presented), treatment of chitosan powder at 180 °C for all time points significantly reduced the endotoxin levels compared to the native chitosan powder, with chitosan treated for 1.5 and 3 h removing endotoxin to levels below FDA-approved limit (Figure 3A). The second method used NaOH for endotoxin removal, as it inactivates endotoxin through hydrolysis of the ester and amide linkages in the lipid A portion of LPS [49,50]. Chitosan powder was treated with 1.0 M NaOH at room temperature for 2 and 24 h, 2 h at 50 °C, and at room temperature for 2 h with the addition of polymyxin B. Polymyxin B was used as it is a high-affinity LPS ligand [51,52], which was postulated to enhance endotoxin removal. All conditions reduced endotoxin contamination of chitosan to below the FDA-approved limit (0.5 EU/mL). There was no significant difference in the level of endotoxin removal between the different conditions (Figure 3B). Chitosan powder heated at 180 °C for 1.5 h and treated with NaOH for 2 h at room temperature were the conditions that advanced to further testing as these were the mildest conditions, assumed to be the least likely to alter the chemical structure of chitosan.

As only chitosan powder incubated in endotoxin-free water was tested in the LAL assay, due to inability to test the dissolved acidic chitosan solution (Section 2.7), a second method was used to confirm endotoxin removal. TNF-α production was measured from PBMCs from three donors cultured with three batches of native, heat-treated and NaOH-treated chitosan films. There was no significant difference in the production of TNF-α between the native chitosan and heat-treated chitosan conditions, suggesting that endotoxin removal through this method was not adequate (Figure 3C). Chitosan solution produced with heat-treated chitosan was inhomogeneous and contained insoluble chitosan flakes (Supplementary Figure S3C), which resulted in a rough film on the surface of the plate. This may have promoted TNF-α release, as a previous study demonstrated that proinflammatory cytokine release from PBMCs treated with biomaterial correlated with surface roughness [58]. However, there was a significant reduction in TNF-α production when PBMCs were cultured on NaOH-treated chitosan films compared to native chitosan films. Taken together, the data suggest that NaOH treatment efficiently removes endotoxin from chitosan and prevents activation of immune cells.

### 3.3. Investigating the Structural Changes of Treated Chitosan

**Figure 4:**
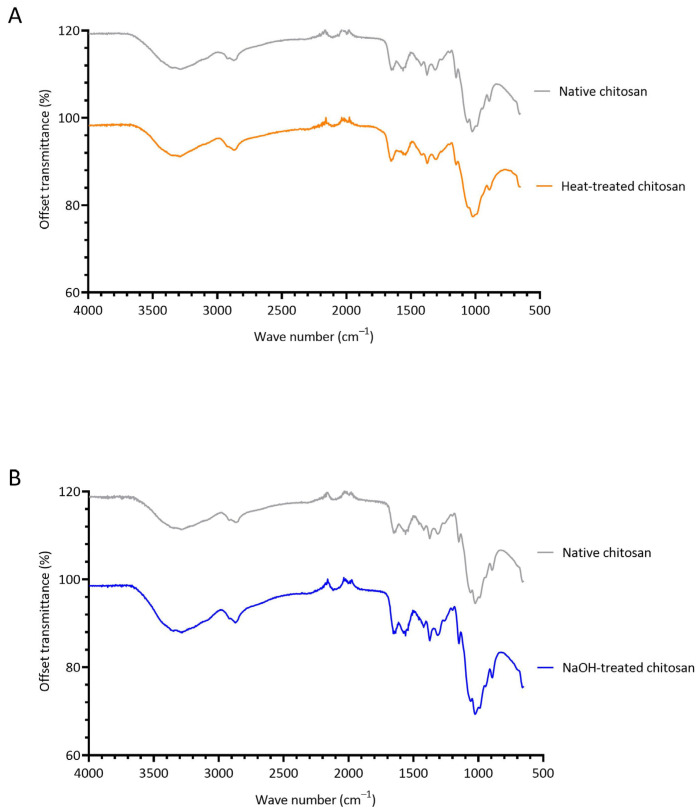
FTIR spectra of native chitosan powder vs. heat-treated chitosan powder (heated at 180 °C for 1.5 h) (**A**) and native chitosan powder vs. NaOH-treated chitosan (treated with 1.0 M NaOH solution for 2 h, washed with endotoxin-free water and dried at 37 °C for 24 h) (**B**).

FTIR spectroscopy was conducted to determine if heat and NaOH-treatment induce chemical changes to native chitosan (Figure 4). In the FTIR spectra of all chitosan samples, a wide band is observed at 3600–3000 cm^−1^, which is due to overlapping O-H and N-H stretching vibrations [59]. A prominent band at approximately 2900 cm^−1^ is present in all samples, which is attributed to C-H stretching of the pyranose ring [9,60]. Characteristic peaks of chitosan are observed at 1647 cm^−1^ and 1559 cm^−1^. The former represents C=O stretching of the secondary amide (amide I band) present in the acetylated units of chitosan, and the latter is due to N-H bending in the secondary amide (amide band II). The band at 1375 cm^−1^ is assigned to CH_3_ bending of the acetylated units of chitosan [61], and the band at 1312 cm^−1^ corresponds to C-N stretching in secondary amides (amide III). The transmission band at 1149 cm^−1^ in the spectra of the chitosan powder is attributed to asymmetric stretching of the C-O-C bridge from the glycosidic bond [62].

Heat treatment caused chitosan powder to darken in colour (Supplementary Figure S3B), which was postulated to be a result of the Maillard reaction (methodology and results can be found in Supplementary Materials [63,64,65,66,67,68,69,70,71,72]). The Maillard reaction is a non-enzymatic browning reaction initiated by the condensation of NH_2_ and C=O groups, resulting in Schiff base formation and rearrangement to Amadori products (Supplementary Figure S4) [66,67]. As chitosan contains both functional groups, it is possible the Maillard reaction can occur in chitosan. Heat treatment also prevented dissolution of chitosan in 1% *v*/*v* acetic acid solution, as insoluble particles were distributed throughout the solution (Supplementary Figure S3C). Despite the obvious visual changes, the FTIR of heat-treated and native chitosan were similar. However, it should be noted that heat treatment causes a change in the peak at 1647 cm^−1^, corresponding to C=O stretching of the secondary amide. In the heat-treated chitosan powder, this peak shifts bathochromically to 1655 cm^−1^ and increases in intensity (Figure 4A). Furthermore, the peak ratio of amide I to amide II bands increases in heat-treated chitosan (Supplementary Table S2). As there is overlap between C=O and C=N [73], it is feasible that these changes are due to the formation of a Schiff base due to the Maillard reaction. Hydrogels were attempted to be formed with heat-treated chitosan and 1% *v*/*v* genipin; however, upon visual inspection, the hydrogels were less robust compared to those formed with native chitosan. Fluorescence studies (Supplementary Methods S1.2.2) showed that heat-treated hydrogels produced less fluorescence compared to native hydrogels (Supplementary Figure S5B), indicating less crosslinking occurred. Overall, heat treatment induced both visual and chemical changes to chitosan, and did not reduce TNF-α production by PBMCs (Figure 3C). It was therefore concluded that heat treatment is not a suitable endotoxin removal method and will not progress for further testing in this study.

The FTIR spectra of native and NaOH-treated chitosan powders are extremely comparable, suggesting that NaOH treatment did not induce any major chemical alterations. There were very slight changes in wavelengths by 1 cm^−1^, for example, a hypsochromic shift occurred in the band from 1647 cm^−1^ to 1646 cm^−1^. It was visually noted that NaOH-treatment enhanced the solubility of chitosan, suggesting that an increase in DD had occurred, improving the hydrophilicity of the polymer. Deacetylation is the process of the removal of acetyl groups from chitin and substitution of amino groups [74]. Chitin that is deacetylated above 50% is defined as chitosan [75]. The DD of native chitosan was estimated as 80.4%, which is in agreement with the Sigma-Aldrich certificate of analysis for the specific batch of chitosan used in this study (see Section 2.1). NaOH treatment resulted in similar DD of 80.3%. This result was anticipated as the NaOH treatment used in this study (1.0 M NaOH for 2 h at room temperature) was extremely mild compared to the severe thermoalkaline conditions of chitin deacetylation [76,77,78,79,80]. Comparable DD was further validated by subsequent successful synthesis of chitosan-genipin hydrogels with NaOH-treated chitosan and 1% *w*/*v* genipin (Section 2.8 and Section 2.9).

### 3.4. Lysozyme Degradation of Chitosan-Genipin Hydrogels Made Using NaOH Treated Chitosan

**Figure 5:**
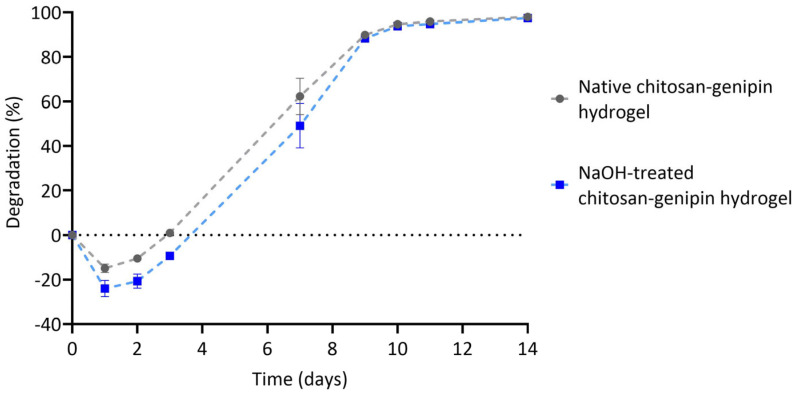
Lysozyme degradation of chitosan-genipin hydrogels composed of native chitosan and NaOH-treated chitosan (treated with 1.0 M NaOH solution for 2 h, washed with endotoxin-free water and dried at 37 °C for 24 h). N = 3.

FTIR results showed that tested NaOH treatment preserved DD of native chitosan. Yet the key to using chitosan as injectable and implantable material is its ability to degrade in vivo. Lysozyme interacts with the acetylated units of chitosan only [4,5], therefore it is important to determine if NaOH-treated chitosan is still susceptible to lysozyme degradation. Native and NaOH-treated chitosan-genipin hydrogel disks were produced following the method outlined in Section 2.9, and lysozyme degradation was monitored gravimetrically. Lysozyme hydrolyses the glycosidic bond in the chitosan backbone [60,81,82] and, as previously shown, also degrades one of the bifunctional crosslinks in chitosan-genipin hydrogels, namely the secondary amide linkage [6]. Figure 5 shows that in the first day of lysozyme exposure, there is a negative degradation rate. This is due to diffusion of the solvent into the network, causing an initial swelling phase [53]. After 1 day, both native and NaOH-treated forms of the gels start to disintegrate, until they are fully degraded in approximately 2 weeks. It is noted that 24 h after the immersion into the solution hydrogels made with NaOH-treated chitosan had a significantly higher volume change (24.0% ± 3.7), compared to native chitosan (15.0% ± 1.9) (*p* < 0.05). This is potentially due to subtle difference in DD and endotoxin removal enhancing hydrophilicity, and therefore diffusion of water into the network. As anticipated, based on similar DD, the degradation rates are comparable; for example, between 3 and 7 days the native and NaOH-treated chitosan-genipin hydrogels degraded by 58.8% and 58.5%, respectively. Overall, the results show that NaOH treatment of chitosan does not affect the lysozyme-mediated degradation of resultant chitosan-genipin hydrogels, making them suitable for in vivo applications and subsequent elimination by degradation.

### 3.5. Compatibility of NaOH-Treated Chitosan-Genipin Hydrogels with moDCs

**Figure 6:**
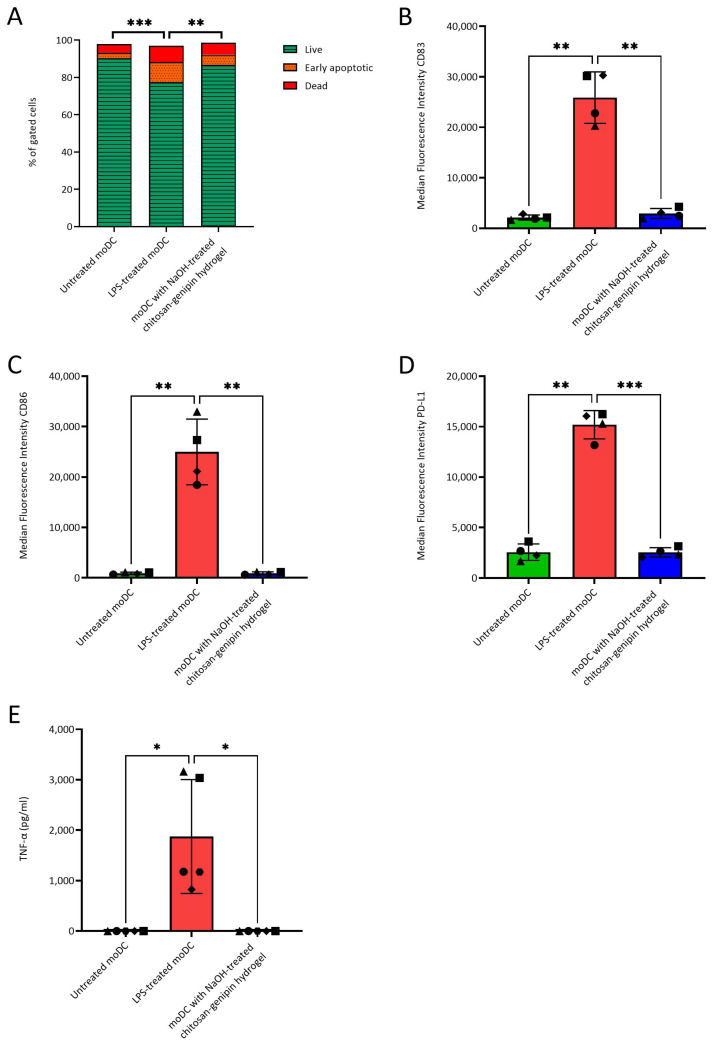
Viability of moDCs cultured without LPS, with 100 ng/mL LPS or with hydrogels made from NaOH-treated chitosan and genipin for 24 h (**A**). Median fluorescence intensity of the maturation markers CD83 (**B**), CD86 (**C**) and PD-L1 (**D**) of moDCs cultured without LPS, with 100 ng/mL LPS or with hydrogels, N = 4. TNF-α production of moDCs cultured without LPS, with 100 ng/mL LPS or with hydrogels (**E**). N = 5 (each symbol represents a different donor). * *p* < 0.05; ** *p* < 0.01; *** *p* < 0.001.

In our previous study with chitosan-genipin hydrogels (identical chitosan used with same lot number sourced from Sigma), we used generic sterilization methods that are not sufficient for endotoxin removal and reported an increase in the release of pro-inflammatory cytokines, IL-6 and TNF-alpha, by murine dendritic cells and monocytes after co-culture with these hydrogels [83]. To investigate the cytocompatibility of NaOH-treated chitosan with immune cells, NaOH-treated chitosan-genipin hydrogels were co-cultured with moDCs. These cells were chosen because they exhibit high sensitivity to endotoxin and are activated by concentrations as low as 0.02 ng/mL LPS (0.1 EU/mL) [22]. Viability, expression of typical DC activation/maturation cell surface markers and secretion of cytokines were tested when moDCs were cultured in the presence of the hydrogels. Untreated and LPS-treated moDCs were used as negative and positive controls, respectively. Whilst LPS treatment significantly reduced moDCs viability compared to untreated moDCs, as expected, co-culture with NaOH-treated chitosan-genipin hydrogels did not affect moDCs viability (Figure 6A). Similarly, LPS treatment significantly increased the expression of moDC cell surface activation/maturation markers and TNF-α secretion, with no significant differences observed between the untreated control and moDCs co-cultured with chitosan-genipin hydrogels (Figure 6B–E). Additional inflammatory cytokines (IL-6, IL-10, IL-12p70 and IL-1β) were also measured and followed a similar trend (Supplementary Figure S6). Overall, these results demonstrate that NaOH-treated chitosan is compatible with this highly endotoxin-sensitive cell type.

## 4. Conclusions

This study demonstrates that endotoxin can be effectively removed from medium molecular weight chitosan by treating powder with 1.0 M NaOH for 2 h at room temperature. The NaOH treatment preserves DD of chitosan and its chemical structure, enabling formation of chitosan-genipin hydrogels, and their subsequent degradation by lysozyme. In vitro studies with moDCs showed that these NaOH-treated chitosan-genipin hydrogels did not affect cell viability, nor induce phenotypical maturation or pro-inflammatory cytokine release. Our results corroborate findings by Lebre and co-workers [41], who successfully employed acid-alkaline treatment of chitosan. Although chitosan is prone to endotoxin contamination, it is not a widely discussed problem in the scientific community. We have evidenced that native chitosan, commonly used in biomaterials research, is contaminated with endotoxin and elicits a proinflammatory response In vitro, which may be the reason behind the vast conflicting reports concerning the immunomodulatory effects of chitosan. As such, endotoxin removal should be a standard procedure, incorporated into methodologies for the synthesis of chitosan-based materials, with NaOH treatment being a simple, inexpensive, and efficient method.

## Data Availability

All data created during this research are openly available at https://doi.org/10.25405/data.ncl.c.6426968 (accessed on 20 March 2023).

## Supplementary Materials

The following supporting information can be downloaded at: https://www.mdpi.com/article/10.3390/polym15071592/s1, Figure S1: Gating strategy for flow cytometry data in Figure 6. Debris (A) and then doublets (B) were first excluded from the cell population. Live, early apoptotic and dead cells were determined based on whether they were stained by Zombie Aqua and Annexin V or not (C). Median Fluorescence Intensity was determined based on the live cell population.; Figure S2: DSC thermograph for chitosan powder subject to 2 heating/cooling cycles and a final heat ramp, which are detailed in Supplementary Methods S1.1 (Top graph, 1st cycle; middle graph, 2nd cycle; bottom graph, 3rd heating ramp).; Figure S3: Appearance of heat-treated chitin and chitosan samples (180 °C for 1.5 hours). (A) Minimal discolouration observed in chitin. (B) Brown discolouration observed in chitosan. (C) Insoluble particles in heat-treated chitosan solution.; Figure S4: Proposed mechanism of the Maillard reaction (extracted from [72]).; Figure S5: The Maillard reaction. (A) The optical density of native chitosan and heat-treated chitosan at 420 nm. (B) Fluorescence intensity of chitosan-genipin hydrogels prepared with native and heat-treated chitosan. N = 3 (black circles indicate repeats). Figure S6: Release of IL-10 (A), IL-12p70 (B), IL-1β (C) and IL-6 (D) cytokines from moDCs cultured without LPS, with 100 ng/ml LPS or with hydrogels. N = 1 (black circles indicate repeats).; Table S1: Flow cytometry antibodies used for surface staining; Table S2: Peak height ratios between amide I and amide II bands in native and heat-treated chitosan powder (180 °C for 1.5 h).
