# Supplementary material for: Effective Endotoxin Removal from Chitosan That Preserves Chemical Structure and Improves Compatibility with Immune Cells
Source: Polymers (Basel). 2023 Mar 23;15(7):1592. doi: 10.3390/polym15071592 (PMC10096541; doi:10.3390/polym15071592)
Supplement: Supplementary file 1 [file polymers-15-01592-s001.zip › polymers-2274542-supplementary.pdf]

polymers

MDPI

Supplementary Materials

Effective Endotoxin Removal from Chitosan That Preserves Chemical Structure and Improves Compatibility with Immune Cells

Sophie L. Reay [1,†], Emma L. Jackson [2,†], Daniel Salthouse [1], Ana Marina Ferreira [1], Catharien M. U. Hilkens [2,*] and Katarina Novakovic [1,*]

[1] School of Engineering, Newcastle University, Newcastle upon Tyne, NE1 7RU, UK
[2] Translational & Clinical Research Institute, Newcastle University, Newcastle upon Tyne, NE2 4HH, UK
* Correspondence: catharien.hilkens@newcastle.ac.uk (C.M.U.H.); katarina.novakovic@newcastle.ac.uk (K.N.)
† These authors contributed equally to this work.

S1. Methods

S1.1 Differential Scanning Calorimetry

Differential scanning calorimetry (DSC) was used to assess thermal transitions and degradation of chitosan powder. DSC was performed using a DSC Q20 (TA instrument) equipped with Universal Analysis 2000 software. Approximately 5 mg of chitosan powder was weighed and sealed in aluminium pans using a Tzero Sample Press Kit. An empty pan was used as reference. The samples were subjected to two heating/cooling cycles and a final heat ramp. In the first cycle for chitosan powder and hydrogels, the samples were heated from -50 °C to 200 °C and cooled to -50 °C. In the second cycle, the samples were heated from -50 °C to 500 °C and immediately cooled down to -50 °C. The final cycle consisted of a single heat ramp from -50 °C to 500 °C. All temperature ramps had a heating/cooling rate of 10 °C/min under constant purging of dry nitrogen at 50 ml/min.

S1.2 Maillard Reaction in Heat-Treated Chitosan

S1.2.1 Optical Density

The browning intensity caused by pigment formation in the final stage of the Maillard reaction can be measured spectrophotometrically at 420 nm. [63,64] Native and heat-treated chitosan (180 °C for 1.5 hours) solutions were prepared following the method outlined in Section 2.4. Chitosan solutions (500 µl) were transferred to Vision Plate™ 24 microplate and optical density at 420 nm was measured using a UV-VIS spectrophotometer (BMG LABTECH, FLUOstar Omega Microplate Reader).

S1.2.2 Fluorescence

The Maillard reaction can also be monitored by measuring the amount of reactive primary amines. The amount of reactive free amino groups in heat-treated chitosan was measured using genipin crosslinking and fluorescence. Chitosan-genipin hydrogel films were prepared following the method outlined in Section 2.8. A UV-VIS spectrophotometer (BMG LABTECH, FLUOstar Omega Microplate Reader) was used to measure the fluorescence intensity (FI) of samples using 550 nm and 650 nm for the excitation and emission wavelengths, respectively, over 24 hours. A bottom-up measurement was used to avoid measurement errors associated with condensation on the plastic cover. A matrix of 10 x 10 data points was scanned with 100 flashes per scan point and 0.2 s settling time. For each well, the average value of all scan points was used to plot FI against time.

S1.3 Flow cytometry antibodies

Table S1. Flow cytometry antibodies used for surface staining.

Antibody	Dilution	Fluorophore	Clone	IsoType	Company	Catalog Number
CD83	1:20	PerCP-Cy5.5	HB15e	Mouse	Biolegend	305320
CD86	1:20	BV711	IT2.2	Mouse	Biolegend	305440
CD274 / PD-L1	1:20	BUV395	MIH1	Mouse	BD Biosciences	740320
Annexin V	1:20	APC	-	Human	Biolegend	640941
**Viability Dyes**	**Concentration**	**Detection**	**Clone**	**IsoType**	**Company**	**Catalog Number**
Zombie Aqua	2ul/ condition	405nm 525/50	-	-	Biolegend	423102

S1.4 FTIR amide ratio

Table S2. Peak height ratios between amide I and amide II bands in native and heat-treated chitosan powder (180 °C for 1.5 h).

Sample	Amide I peak height (C=O or C=N stretching) (%)	Amide II peak (N-H bending) height (%)	Peak height ratio amide I: amide II
**Native chitosan**	8.98	9.18	1: 1.02
**Heat-treated chitosan**	9.84	8.47	1: 0.86

S1.2.5 Multiplex cytokine analysis

Cytokines from cellular supernatant of moDCs was measured using U-PLEX® Custom Biomarker (hu) Assays, Meso Scale Discovery (Rockville, MD, USA) according to manufacturer's protocol. Plates were read on MESO QuickPlex SQ 120 (Meso Scale Discovery).

S2 Results

S2.1 The Maillard reaction

Heat treatment caused chitosan powder to darken in colour, in which the colour intensity was proportional to the temperature and time of the treatment (Supplementary Figure 3B). This trend was also observed by Yang *et al.* who steam-heated chitosan powder. [65] It was postulated that the discolouration was due to the Maillard reaction, which is a non-enzymatic browning reaction. The Maillard reaction is initiated by the condensation of NH_2 and C=O groups, resulting in Schiff base formation and rearrangement to Amadori products (Supplementary Figure S4). [66,67] As chitosan contains both functional groups, it is possible the Maillard reaction can occur in chitosan. Two tests were conducted to determine if the Maillard reaction took place in the heat-treated chitosan. The browning intensity caused by pigment formation in the final stage of the Maillard reaction can be measured spectrophotometrically at 420 nm. [63,64,68] Supplementary Figure S5A shows that the optical density of optimised heat-treated chitosan at 420 nm is significantly higher than that of native chitosan ($p = <0.0001$), with the degree of browning approximately 11 times greater. Heat-induced discolouration was also tested in chitin, which does not contain amino groups (Sigma do not disclose the degree of acetylation of chitin, therefore it may not be 100%), where minimal colour change occurred (Supplementary Figure S3A). This suggests that the colour change is attributed to the amino groups present in chitosan.

The Maillard reaction can also be monitored by measuring the amount of reactive amino groups, for example, amino groups react with O-phthalaldehyde to produce a fluorescent product that can be measured spectrophotometrically. [69] In this study, a novel approach was used to determine whether the Maillard reaction took place by assessing

the amount of reactive free amino groups, using genipin crosslinking and fluorescence. Genipin reacts with primary amine groups of chitosan, leading to the production of highly conjugated heterocyclic fluorescent products. [70,71] Fluorescence intensity therefore correlates with the number of primary amine groups available for genipin crosslinking. Fluorescence studies showed that hydrogels synthesised with heat-treated chitosan produced significantly less fluorescence at 24 hours compared to those composed of native chitosan (p = <0.0001) (Supplementary Figure S5B). This indicates that heat treatment reduced the number of free amino groups available for genipin crosslinking, suggesting that the Maillard reaction took place.

S3 Figures

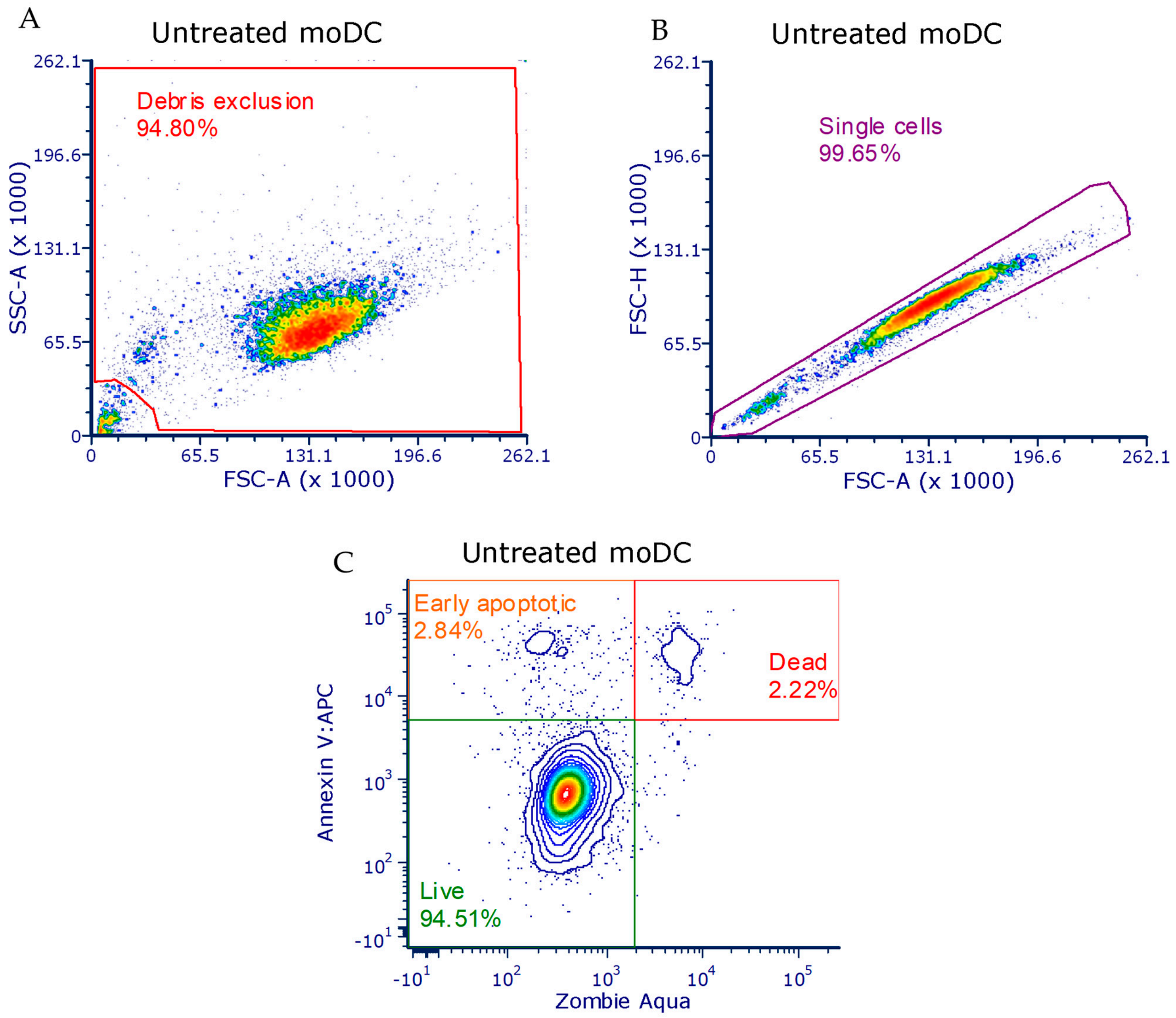

Figure S1. Gating strategy for flow cytometry data in Figure 6. Debris **(A)** and then doublets **(B)** were first excluded from the cell population. Live, early apoptotic and dead cells were determined based on whether they were stained by Zombie Aqua and Annexin V or not **(C)**. Median Fluorescence Intensity was determined based on the live cell population.

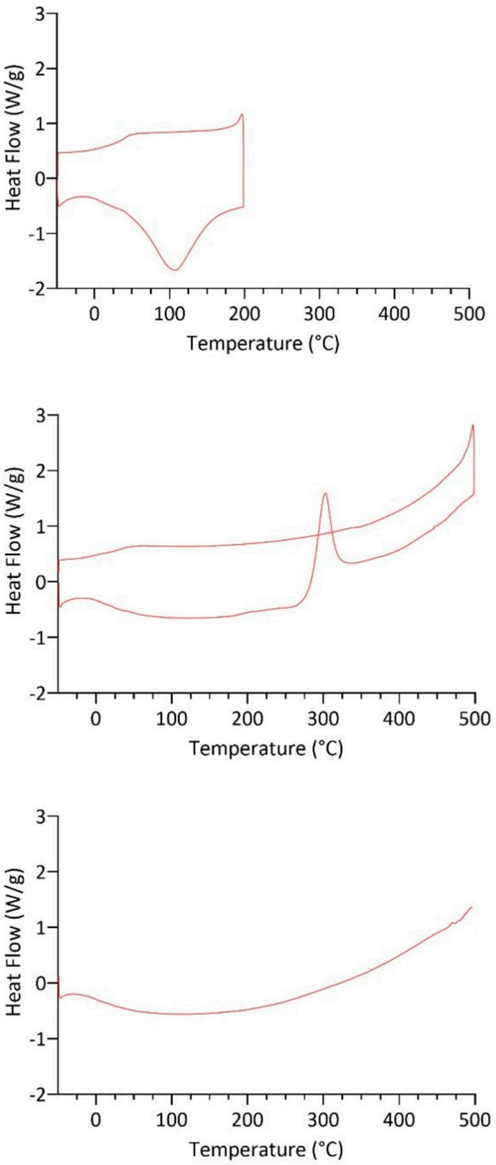

Figure S2. DSC thermograph for chitosan powder subject to 2 heating/cooling cycles and a final heat ramp, which are detailed in Supplementary Methods S1.1 (Top graph, 1st cycle; middle graph, 2nd cycle; bottom graph, 3rd heating ramp).

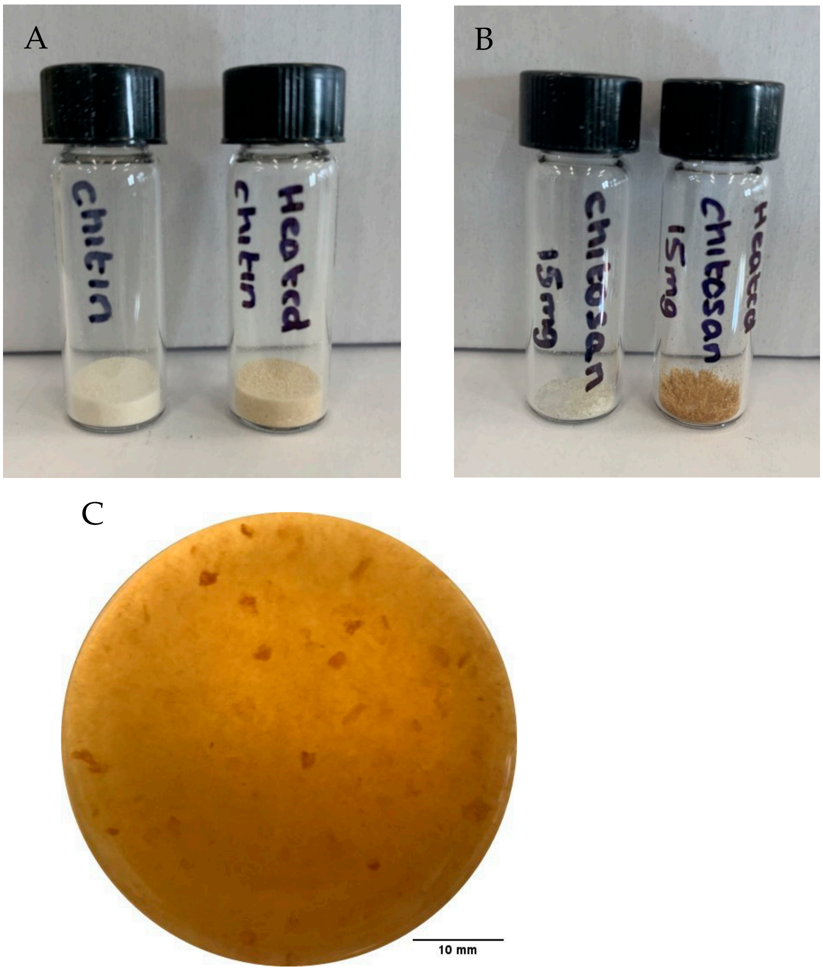

Figure S3. Appearance of heat-treated chitin and chitosan samples (180 °C for 1.5 hours). **(A)** Minimal discolouration observed in chitin. **(B)** Brown discolouration observed in chitosan. **(C)** Insoluble particles in heat-treated chitosan solution.

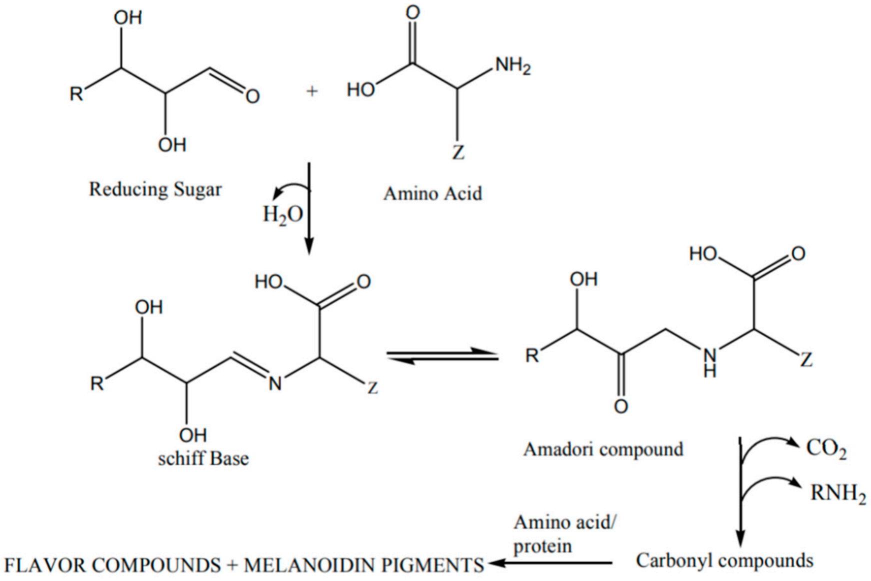

Figure S4. Proposed mechanism of the Maillard reaction (extracted from [72]).

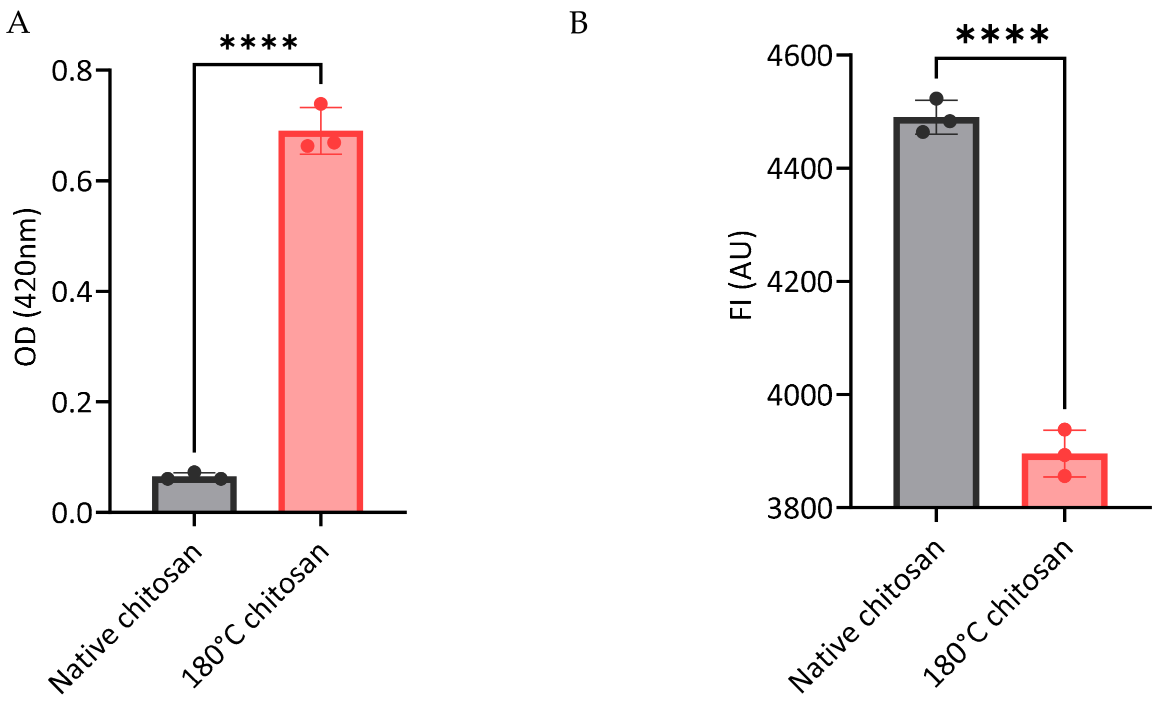

Figure S5. The Maillard reaction. **(A)** The optical density of native chitosan and heat-treated chitosan at 420 nm. **(B)** Fluorescence intensity of chitosan-genipin hydrogels prepared with native and heat-treated chitosan. N=3 (black circles indicate repeats).

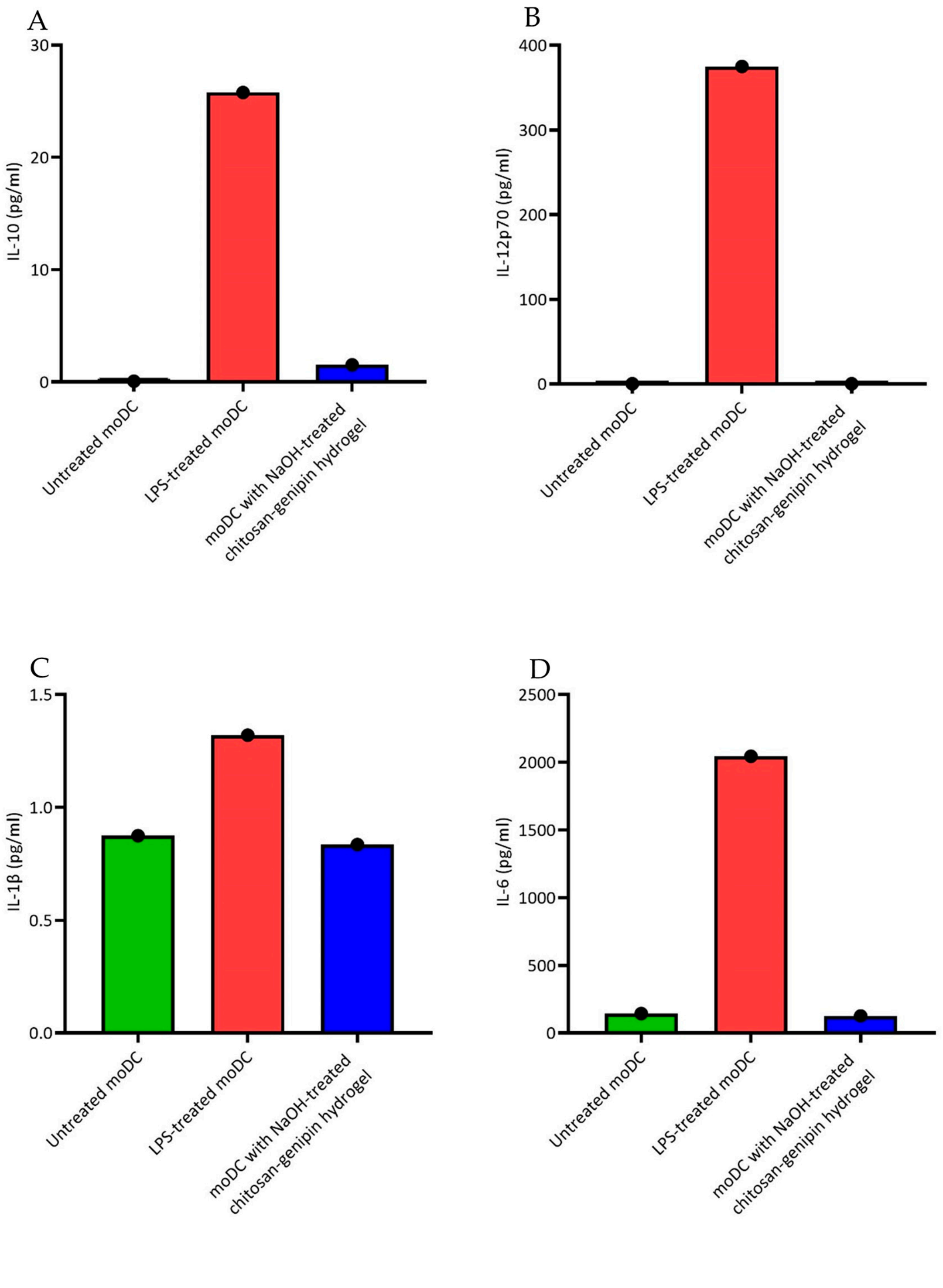

Figure S6. Release of IL-10 **(A)**, IL-12p70 **(B)**, IL-1β **(C)** and IL-6 **(D)** cytokines from moDCs cultured without LPS, with 100 ng/ml LPS or with hydrogels. N=1 (black circles indicate repeats).
